# Consumer involvement at the MRC Clinical Trials Unit: results of a survey

**DOI:** 10.1186/1745-6215-12-S1-A81

**Published:** 2011-12-13

**Authors:** Claire L Vale, Lindsay C Thompson, Silvia Forcat, Claire Murphy, Bec Hanley

**Affiliations:** 1 MRC Clinical Trials Unit, London, WC2B 6NH, UK

## Background

We aimed to establish levels of consumer involvement in randomised controlled trials (RCTs), meta-analyses and other studies carried out by the MRC Clinical Trials Unit across the range of research programs, predominantly in cancer and HIV.

## Methods

Staff responsible for studies included in a Unit Progress Report (MRC CTU, April 2009) were asked to complete a questionnaire survey regarding consumer involvement. This was defined as active involvement of consumers as partners in the research process and not as subjects of that research. The electronic questionnaires combined open and closed questions, intended to capture quantitative and qualitative information on whether studies had involved consumers; types of activities undertaken; recruitment and support; advantages and disadvantages of involvement and its perceived impact on aspects of the research.

## Results

Between October 2009 and April 2010, 138 completed questionnaires (86%) were returned. Studies had been conducted over a 20 year period from 1989, and around half were in cancer; 30% in HIV and 20% were in other disease areas including arthritis, tuberculosis and blood transfusion medicine. Forty-three studies (31%) had some consumer involvement, most commonly as members of trial management groups (TMG) [88%]. A number of positive impacts on both the research and the researcher were identified. Researchers generally felt involvement was worthwhile and some felt that consumer involvement had improved the credibility of the research. Benefits in design and quality, trial recruitment, dissemination and decision making were also perceived. Researchers felt they learned from consumer involvement, albeit that there were some barriers. Additional results will be presented.

## Conclusions

Whilst most researchers identified benefits of involving consumers, most of studies included in the survey had no involvement. Information from this survey will inform the development of a unit policy on consumer involvement, to guide future research conducted within the MRC Clinical Trials Unit.

